# Controlling evolution of protein corona: a prosperous approach to improve chitosan-based nanoparticle biodistribution and half-life

**DOI:** 10.1038/s41598-020-66572-y

**Published:** 2020-06-15

**Authors:** Farnaz Sadat Mirzazadeh Tekie, Maliheh Hajiramezanali, Parham Geramifar, Mohammad Raoufi, Rassoul Dinarvand, Masoud Soleimani, Fatemeh Atyabi

**Affiliations:** 1 Nanotechnology Research Centre, Faculty of Pharmacy, Tehran University of Medical Sciences, Tehran, Iran; 2 Department of Radipharmacy, Faculty of pharmacy, Tehran University of Medical Sciences, Tehran, Iran; 3 Research Center for Nuclear Medicine, Shariati Hospital, Tehran University of Medical Sciences, Tehran, Iran; 4 Department of Pharmaceutical Nanotechnology, Faculty of pharmacy, Tehran university of medical sciences, Tehran, Iran; 5 Department of Pharmaceutics, Faculty of Pharmacy, Tehran University of Medical Sciences, P.O. Box 14155-6451, Tehran, Iran; 6 Department of Molecular Biology and Genetic Engineering, Stem Cell Technology Research Center, P.O. Box 14155-3174, Tehran, Iran; 7 Department of Hematology, School of Medical Sciences, Tarbiat Modares University, P.O. Box 14115-111, Tehran, Iran

**Keywords:** Polysaccharides, Drug delivery

## Abstract

Protein corona significantly affects *in vivo* fate of nanoparticles including biodistribution and half-life. Without manipulating the physicochemical properties of nanoparticles with considering their biointerference, attaining effective treatment protocols is impossible. For this reason, protein corona evolution and biodistribution of different chitosan (Ch)-based nanoparticles including Ch and carboxymethyl dextran (CMD)/thiolated dextran (TD) polyelectrolyte complexes (PECs) were studied using highly precious and sensitive methods such as liquid chromatography-mass/mass (LC-MS/MS) spectroscopy and positron emission tomography/computed tomography (PET/CT) scan. The importance of serum presence/absence in culture medium with different pH and corona effect on cellular uptake of PECs investigated by *in vitro* study. Designed PECs have low amounts of proteins in corona mostly enriched by Apolipoproteins, protein C, hemoglobin subunits, and inter-alpha- trypsin inhibitor that beside improving uptake of nanoparticles, they have low liver uptake and notable heart blood pool accumulation that confirmed the long circulation time of the nanoparticles which is favorable for delivery of nanoparticles to the site of action and achieving required therapeutic effect.

## Introduction

It was declared by many researches that biodistribution and cell internalization of nanoparticles is extremely affected by protein corona^1,2^, which depends on nanoparticle properties such as surface charge, hydrophobicity, presence of ligands^3,4^, size, and morphology^5–7^; medium composition such as protein source^8^; medium condition such as pH^9^; and exposure time^10,11^. It was asserted that altering of protein configuration after adsorption to the nanoparticles, and consequent exposure of some epitopes, which are naturally buried in the interior sites of proteins, initiate the immune response by making the nanoparticles recognizable for phagocytes. This phenomenon, opsonization, causes rapid clearance of nanoparticles from plasma and low absorption to target sites; instead they mostly accumulate in liver and spleen. Here, not only amount of adsorbed protein, but also type and configuration of them must be considered since they determine the fate of nanoparticles by opsonization^10,12,13^. To attain longer half- life for nano scale delivery systems, the most prevalent strategy is to cover the surface of nanoparticles by some polymers such as polyethylene glycol (PEG)^14–16^, poloxamer^17^, and dextran^18,19^. Gref *et al*. fabricated a series of PEG-coated poly lactic acid (PLA), poly (lactic-co-glycolic acid) (PLGA) and poly ε-caprolactone (PCL) nanoparticles with different molecular weights (Mw) of PEG, and showed that adsorption of plasma proteins to the nanoparticles depends on PEG size and content^20^. Natte *et al*. prepared core-shell structured nanoparticles composed of silica as a core and PEG as a shell. They demonstrated that increasing Mw of PEG suppresses protein corona formation. Furthermore, they affirmed dynamical evolution of protein corona during incubation with serum^21^. Dextran was also used as a coating to improve efficacy of drug delivery systems^22,23^. Dextran salts such as dextran sulfate and carboxymethyl dextran (CMD) was developed into colloidal polyelectrolyte complexes (PECs) in a company of chitosan (Ch), a natural cationic polysaccharide^24–27^. Ch based nanoparticles are tremendously used as gene and drug delivery systems^28,29^. Nonetheless, plasma proteins adsorbed to the polymer by electrostatic, and hydrogen bonding due to the cationic nature of the polymer and presence of functional amine groups on deacetylated Ch, consequently interfere with nanoparticle function and circulation *in vivo*^30,31^. Constructing Ch nanoparticles covered with hydrophilic polymers such as PEG enhances their half-life in serum^30,32^. Obviously, instead of preparing copolymers of Ch and hydrophilic polymers, it is easier and safer to produce PECs since they obtained in aqueous solution by electrostatic interactions between opposite charges of polymers needless to the further toxic reagents or harmful condition. Lin *et al*. prepared the PECs of CMD and Ch and indicated the stability of formulation containing sugar during storage time for more than one week, and the stability of PECs during autoclave procedure^27^. Also they showed prolonged release profile of a drug from the PECs^33^.

In our pervious study, the nano PECs of CMD and Ch were introduced as gene delivery systems. It was demonstrated that Ch Mw and dextran to Ch molar ratio (D/Ch) affect the nanoparticle physical properties and *in vitro* efficacy while there was an interaction between effects of these parameters. It was found that by increasing Ch Mw, the PECs with higher D/Ch ratios are more effective, probably due to the adequate balance between their serum stability and cytoplasmic dissociation rate^34^.We also compared the thiolated PECs composed of Ch/thiolated Ch and CMD/thiolated dextran (TD) with the non-thiolated ones indicating that thiolated PECs have more stability and higher transfection efficacy^35,36^. It was established that protein corona influences the fate of nanoparticles and Ch based nanoparticles are so attractive in field of gene/drug delivery, hence a comprehensive research on protein corona evolution with Ch nanoparticles seems necessary^37^.

In this study, we investigated the protein corona formation of the Ch based PECs in serum which could affect transfection efficacy and *in vivo* biodistribution of the system. The PECs of Ch and CMD or TD with various Ch Mw and D/Ch ratios were prepared to investigate effects of the structural parameters on protein corona evolution. Furthermore, *in vitro* studies on the breast cancer cell line (MCF-7) were performed to compare uptake and toxicity of the nanoparticles with various structures in presence and absence of serum in culture mediums with different pH. Finally, *in vivo* study was accompanied to evaluate the biodistribution of the various PECs and possible correlation between protein corona and biodistribution.

## Materials and methods

### Materials

Supporting Information S1.

### Synthesis and characterization of polymers

#### Synthesis and purification of TD

TD was synthesized by the covalent linkage of L-Cys to CMD via amide bonds involving the primary amine groups of L-Cys and carboxylic acid groups of CMD as described by Shahnaz *et al* with minor modification explained in Supporting Information S2 ^38^.

#### Low Mw Ch preparation and characterization

Chitosan with low Mw was prepared as described in Supporting Information S3 ^34^.

### Fabrication and characterization of PECs

The PECs of Ch9 or Ch18 and TD or CMD with various compositions (Table 1) were prepared. Adequate volume of CMD or TD stock solution (2 mg/ml in acetate buffer; pH, 5.5) was diluted with adequate amounts of the buffer. Afterward, sufficient amount of Ch in acetate buffer solution (pH, 5.5) was added and mixed by several pipetting up and down. The final volume of the formulations was 0.5 ml. Subsequent to 20 s intense vortex stirring, the mixtures were stored at room temperature for about 30 min.

**Table 1:** The formulations of various PECs.

^a^D/Ch	^b^Ch (µg/ml)	^c^CMD or ^d^TD (µg/ml)
0.2	558	120
1	282	302
5	90	480

^a^D/Ch: dextran to chitosan molar ratio.

^b^Ch: chitosan.

^c^CMD: carboxtmethyl dextran.

^d^TD: thiolated dextran.

Physical characterization of nanoparticles including thermal gravimetric analysis (TGA), transmission electron microscopy (TEM) and evaluation of size distribution (diameter and poly dispersity (PDI)) and zeta potential by dynamic light scattering (DLS) were accomplished on freshly prepared PECs as described in Supporting Informations S4–S6.

### PEC structure influence on protein corona formation

#### Evolution of protein corona

FBS with a concentration of 30% (v/v) was added to the 500 µl of prepared PECs containing 100 µg of nanoparticles, and the samples were incubated at 37 ^o^C for 1 h. To remove the soft corona and evaluating the hard corona, the incubated PECs were separated by centrifuging at 13000 rpm and washed two times by adding 1 ml of phosphate buffer solution (PBS, pH 7.4) and centrifuging at 15000 rpm for 30 min^39^. The nanoparticles were dispersed in PBS (pH 7.4) for further analysis.

#### Effect of protein corona on size and zeta potential of the PECs

Size distribution and zeta potential of the PECs were determined subsequent to protein corona formation in FBS before and after removing soft corona.

#### SDS-page electrophoresis

The hard corona was investigated using acrylamide SDS-page electrophoresis. Following formation of protein corona, washing and dispersing the PECs in PBS, the proteins interacted with nanoparticles was eluted using SDS-sample buffer and boiled for 5 min at 100 ^o^C. The samples were then investigated by 1D gel 12% Acrylamide SDS- page electrophoresis^40^.

#### Liquid chromatography –Mass/Mass (LC-MS/Ms) spectroscopy

LC-Ms/Ms was performed to determine protein corona construction, spectral count of peptides (SPC) and relative quantity of each protein emerged on PEC corona as described in Supporting Information (S7)^41,42^.

#### Circular dichroism (CD) spectroscopy

It was explained in Supporting Information S8.

### *In vitro* study

#### Design of *in vitro* tests

Influence of the nanoparticle formulation variables including Ch Mw, kind of dextran (TD or CMD), D/Ch ratio, pH of the culture medium, and presence of FBS in culture medium were studied on uptake and toxicity of the PECs on MCF7 cell line, as it is summarized in Table 2.

**Table 2:** Design of *in vitro* experiments.

Test NO.	^a^CMD, ^b^TD, or ^c^DNA	^d^Ch Mw (KD)	^e^D/Ch	^f^FBS %(v/v)	Medium pH
1	TD	18	0.2	10	7.4
2	CMD	18	5	10	7.4
3	TD	9	5	10	7.4
4	TD	18	5	10	7.4
5	TD	18	0.2	10	6.8
6	CMD	18	5	10	6.8
7	TD	9	5	10	6.8
8	TD	18	5	10	6.8
9	TD	18	0.2	0	7.4
10	CMD	18	5	0	7.4
11	TD	9	5	0	7.4
12	TD	18	5	0	7.4
13	TD	18	0.2	0	6.8
14	CMD	18	5	0	6.8
15	TD	9	5	0	6.8
16	TD	18	5	0	6.8
*17	DNA	18	—	10	7.4
*18	DNA	18	—	10	6.8
*19	DNA	18	—	0	7.4
*20	DNA	18	—	0	6.8

^a^CMD: arboxymethyl dextran

^b^TD: thiolated dextran

^c^DNA: deoxyribonucleic acid

^d^Ch: chitosan

^e^D/Ch: the molar ratio of dextran to chitosan

^f^FBS: fetal bovine serum

^*^These tests were carried out using the polyplexes composed of Ch and the Cy5 conjugated oligonucleotide with Ch amine to DNA phosphate group ratio (N/P) of 40.

#### Preparation of fluorescent-labeled PECs

The Cy5 conjugated scramble oligonucleotide (Cy5-oligo) was incorporated into the nanoparticles in a middle of PEC preparation procedure by adding to the dextran solution followed by complexation with Ch as described in sec. 3.3.1.

In order to prepare the PECs of Ch18 and Cy5-oligo with N/P (amine of Ch to phosphate of DNA molar ratio) of 40, the oligonucleotide was mixed with Ch in acetate buffer solution (pH, 5.5) using vigorous vortex stirring (2500 rpm) for about 20 s followed by incubating at room temperature for 30 min.

#### The *in vitro* cellular studies

These experiments are explained in Supporting Information S9–S11 ^43^.

### *In vivo* biodistribution

#### Preparation of ^68^Ga-labeled nano PECs

p-SCN-Bn-DOTA (2.4 mg) was added to the Ch 18 KD suspension in carbonate buffer (pH, 9) followed by stirring with 1200 rpm at room temperature for 36 h. Purification of chitosan- p-SCN-Bn-DOTA (Ch-DOTA) conjugate was performed by 500- 1000 Da dialysis tube (spectrum labs, USA) in distilled water.

The generator was eluted with 0.5 M HCl solution. 150 µl Ch-DOTA (1.5 mg/ml) was mixed with 2 ml collected fraction (9 mCi activity) which its pH had been adjusted to 4.5 by NaOH solution 0.6 M. The mixture was incubated at 90 ^o^C for 5 min. The unbounded ^68^Ga was separated from solution using Amicon® centrifugal filter (10 KD cutoff).

The yield of radio labeling before and after purification was determined using thin layer chromatography (TLC) on Whatman® paper No. 3 (Sigma Aldrich, USA) and 100 mM sodium citrate solution as a mobile phase. The unbounded ^68^Ga was evaluated by TLC scanner (MiniGita, Elysia-Raytest, Germany).

The radio labeled nano PECs was prepared by the method described in sec. 3.3.1 and the formulations presented in Table 1. The amount of Ch-DOTA was 45 µg/ml in each formulation which was subtracted from Ch quantity in formulation to kept Ch molarity constant.

#### Stability of radio labeled nano PECs

The stability of radio labeled nano PECs was investigated in human serum by incubating the mixture of nano PECs and human serum with volume ratio of 1:10 at 37 ^o^C. Every 30 min, the aliquot was evaluated by TLC on RP-18 modified silica gel TLC plate (EMD Millipore, Massachusetts, USA) and sodium citrate solution (100 mM) as the mobile phase.

#### Positron emission tomography/computed tomography (PET/CT) study

*In vivo* study was performed to compare the biodistribution of the PECs composed of Ch and CMD with D/Ch ratio of 5, Ch and TD with D/Ch ratio of 0.2 and 5. The animal experiments were carried out, as explained in Supporting Information S12.

All methods were carried out in accordance with guidelines of ethical care and use of research animal at Tehran University of medical sciences and all experimental protocols were specifically approved by the Committee of Ethics of the Faculty of Sciences of Tehran University (No. 357).

### Statistical analysis

All shown data are the mean of at least 4 replications minus-plus standard deviation (SD). One-way analysis of variance (ANOVA) was performed to evaluate and compare the obtained results while a difference with p-value <0.05 were considered significant.

## Results and Discussion

### Polymer preparation and characterization

The results were provided in Supporting Information S13.

### PEC preparation and characterization

Following preparation of the polymers, the PECs were fabricated by electrostatic interactions between carboxyl moieties of CMD and TD and quaternary amine groups of Ch via coacervation complexation. By considering pKa of Ch amine groups (pKa 6.5), CMD carboxymethyl moieties (pKa 4), and Cys carboxylic acid groups (pKa 2.05), both Ch and dextran are ionized at selected pH (pH 5.5) in aqueous medium. Therefore, nanoparticles were simply developed because of interactions between positive and negative charges of the polymers by vigorous vortex stirring of formulations for a short time. In case TD is used in the formulations, both electrostatic interactions and disulfide bonds between TD chains contribute in nanoparticle configuration and stabilization. The PECs with various surface properties were obtained using CMD or TD, and altering D/Ch molar ratio. Surface properties of nanoparticles are the crucial factors that determine the biointerference phenomenon^44^. TGA confirmed complexation of CMD and Ch. The obtained results were reported by details in Supporting Information (S14). As illustrated in Fig. S2, weight loss of PECs due to the CMD or Ch decomposition (90.364%) occurred with T_peak_ of 286.26 ^o^C which is between T_peak_ of Ch and CMD demonstrating complexation of the polymers by electrostatic interaction^45^.

**Figure 1:**
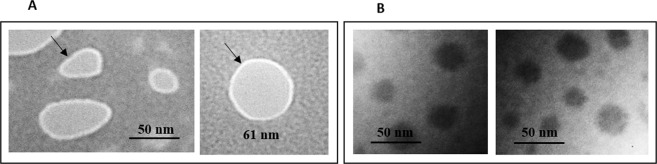
Transmission electron microscopy images of PEC composed of TD and Ch18 with D/Ch ratio of 5 (**A**), and 0.2 (**B**). The arrows indicated the dextran enriched shell of nanoparticles.

The nanoparticle morphology was investigated using TEM. As shown in Fig. 1, the nano PECs possesses semi spherical shape. Despite of thiolated PECs with D/Ch ratio of 0.2, the PECs with D/Ch ratio of 5 did not have homogenous structure and it seems they have a dense core and a soft shell. The size of nanoparticles depends on their structure characterized 100 ± 12 nm and >20 nm for D/Ch ratio of 5 and 0.2 respectively. The thickness of heterogeneous dextran enriched shell of PECs with D/Ch ratio of 5 was estimated 2-10 nm.

The PEC zeta potential and size distribution determined by zeta sizer and DLS were presented in Table 3 which is in acceptable accordance with TEM results. The size of nanoparticles was similar to the estimated size obtained from TEM but because of swelling of PECs in aqueous medium, the hydrodynamic size was slightly higher. The negative zeta potential of the PECs with D/Ch ratio of 5 is because of the dextran enriched shell of the nanoparticles observed in the TEM image. The initial size of PECs could be influenced by parameters manipulating nanoparticle composition such as kind of dextran (CMD/TD), Ch Mw, and D/Ch ratio. Moreover, PECs are kind of nanohydrogels which absorb water when disperse in liquid medium and the hydrodynamic size increases following swelling of nanohydrogels^34^. The results of DLS test was further discussed in Supporting Information S15.

**Table 3:** Physical characteristics of the PECs before and after incubation in FBS.

D/Ch	Ch Mw (KD)	CMD or TD	S1 (nm)	PDI	ɀ (mv)	S2/S1	S3/S1	Hɀ
0.2	9	TD	70.0 ± 3.2	0.26 ± 0.03	18.8 ± 1.9	5.22	3.59	−12.5 ± 2.8
1	9	TD	128.0 ± 8.6	0.26 ± 0.03	7.68 ± 1.8	2.28	1.28	−4.5 ± 2.3
5	9	TD	142.0 ± 7.7	0.17 ± 0.02	−9.31 ± 1.3	2.08	1.18	−16.9 ± 3.5
0.2	18	TD	26.5 ± 2.3	0.39 ± 0.04	21.2 ± 1.2	8.59	3.61	−3.4 ± 1.2
1	18	TD	34.0 ± 3.7	0.21 ± 0.05	20.8 ± 2.3	3.86	3.47	−2.8 ± 1.7
5	18	TD	56.0 ± 4.0	0.12 ± 0.04	−1.69 ± 0.7	1.37	1.14	−13.6 ± 3.6
0.2	9	CMD	30.0 ± 4.7.0	0.20 ± 0.03	16.3 ± 2.3	5.93	4.23	−11.2 ± 3.2
1	9	CMD	217.0 ± 15.0	0.13 ± 0.02	10.2 ± 1.5	2.72	1.15	−8.9 ± 2.8
5	9	CMD	136.0 ± 8.5	0.06 ± 0.02	−11.9 ± 1.0	1.48	1.12	−11.0 ± 2.5
0.2	18	CMD	30.0 ± 2.2	0.30 ± 0.04	18.8 ± 1.6	7.84	3.8	−12.0 ± 1.7
1	18	CMD	190.0 ± 12.0	0.09 ± 0.01	11.8 ± 1.4	2.61	1.75	−7.6 ± 1.9
5	18	CMD	151.0 ± 5.0	0.05 ± 0.02	−11.1 ± 2.1	1.34	1.12	−1.0 ± 2.6

S1: initial size of PECs

PDI: polydispersity index

ɀ: initial zeta potential

S2/S1: the ratio of the size of PECs in serum (soft corona) to the initial size of them

S3/S1: the ratio of the size of PECs with hard corona to the initial size of them

Hɀ: zeta potential of PECs in presence of hard corona

### Effect of nanoparticle structure on protein corona

When the PECs were incubated in serum contained medium, interaction of serum proteins with nanoparticles led to the increase in hydrodynamic size of PECs, count on their structure. Nanoparticle attached proteins are in equilibrium with free proteins in medium before centrifugation and washing process. This equilibrium does not achieve immediately. At first, proteins with high concentrations and association rate constants cover the nanoparticles. The attached proteins are replaced gradually by proteins with lower concentrations and association rate constants, but with higher affinity^6^.

Protein corona is mainly affected by nanoparticle properties, particularly the surface structure. Many parameters such as size, zeta potential, and presence of functional groups on surface of particles determine their biological interactions^7^. In this study, the various PECs were prepared and the formation of protein corona in presence of FBS was investigated.

#### Size and zeta potential of the PECs following corona evolution

One of the important parameters in drug delivery by nanoparticles which influences their biodistribution is the size of nanoparticle in body fluids. Table 3 demonstrates the size of nanoparticles in the formulation medium and the alteration of PEC size in FBS before (soft corona) and after washing by PBS (hard corona).

As shown in Table 3, the noticeable change in size of the PECs with D/Ch ratio of 0.2 was observed, but only the slight change was distinguished in size of the PECs with D/Ch ratio of 5. This phenomenon support our hypothesis that dextran can reduce the interaction of nanoparticles with serum proteins. However, other structural parameters including difference in zeta potential of the particles and deionization of Ch amine groups in FBS pH must be considered as well. For instance, the nanoparticles with D/Ch ratio of 5 mostly contain carboxylate groups and possess the negative charge which even increases in serum condition due to Ch deionization. Such negative zeta potential could inhibit the aggregation of the PECs in serum rather than protein corona formation which was also reduced due to increase the repulsion between nanoparticle and negative charge proteins.

Although, electrostatic interaction has a crucial role in biointerference; we suppose that here it has only a negligible effect since serum proteins have negative charge at physiological pH, and considering the chitosan pKa of 6.8, the PECs also have negative or neutral charges. Therefore, the utmost of interferences occurred with hydrophobic, hydrogen or covalent interactions.

It seems that the non-thiolated PEC size alteration was less than thiolated ones. We assume that oxidation of thiol residues and emerging of disulfide bonds increases the nanoparticle aggregation in serum condition including pH, 7.4 and presence of ions catalyzing thiol oxidation reaction. Thus, increase in hydrodynamic size of thiolated PECs may be due to the aggregation of the nanoparticles by disulfide bridges between them in serum. Additionally, the thiolated PECs could interact with serum proteins which consist of amino acids containing thiol groups such as cysteine via disulfide bonds that lead to the increase in size and further aggregation of the particles^46^.

In spite of D/Ch ratio, Ch Mw had no significant effect on size alteration in FBS.

The loosely bound proteins de-attached by separation, washing and re-dispersion of nanoparticles. The proteins that still remain on the surface of nanoparticles developed hard corona^6^. All samples, the nanoparticle size was decreased after washing. Higher amounts of dextran obviously diminished the hard corona around PECs. The hard corona zeta potential of PECs was negative in PBS because of the negative charges of the attached proteins and deionization of chitosan at pH 7.4.

It must be considered that acetate buffer, FBS, and PBS are different media which affect the PEC characteristics such as their size, swelling, aggregation and zeta potential. Furthermore, centrifuging of chitosan based PECs led to the non-reversible nanoparticle aggregations. To have a confident conclusion about effects of the particle structure on formation of protein corona around the PECs, the more robust tests including SDS-page electrophoresis and LC-MS/MS spectroscopy were performed.

#### SDS-page electrophoresis

**Figure 2:**
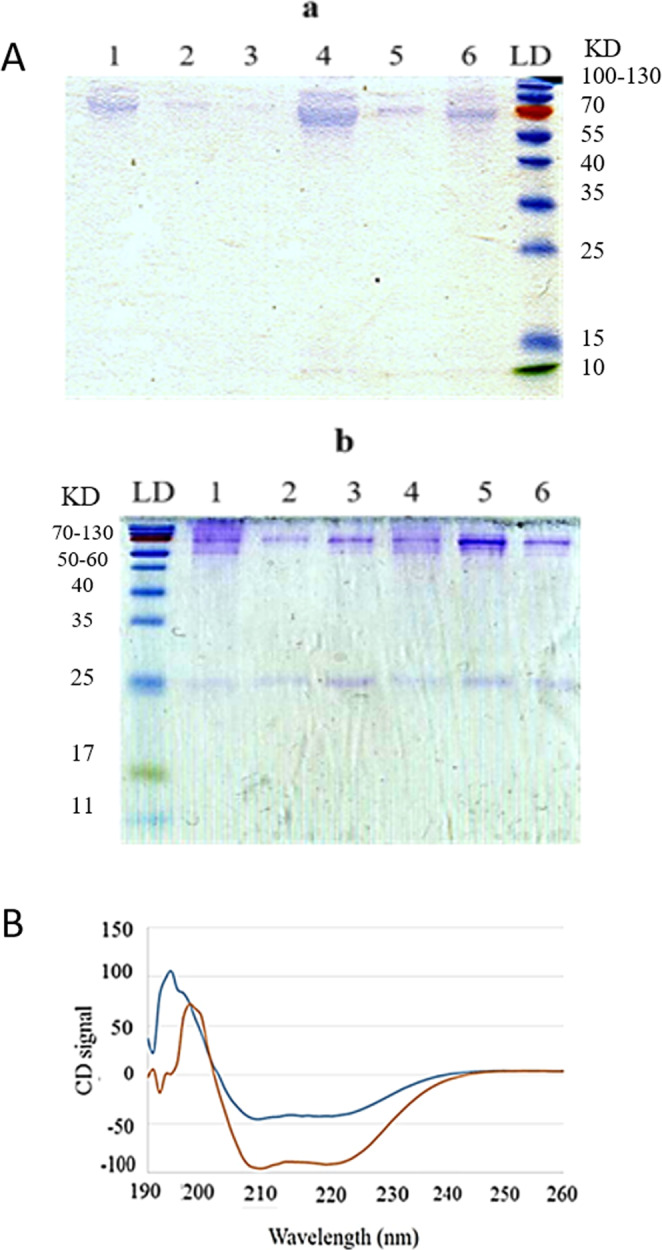
Protein corona evaluation; (**A**) Acrylamide-SDS page electrophoresis. Gel A shows the samples composed of TD and Gel B shows the ones composed of CMD. PECs of Ch9 with D/Ch ratio of 0.2 (1), with D/Ch ratio of 1 (2), with D/Ch ratio of 5 (3), PECs of Ch18 with D/Ch ratio of 0.2 (4), with D/Ch ratio of 1 (5), with D/Ch ratio of 5 (6). The unedited figure is shown in Fig. S3. (**B**) CD spectra of HSA before (red line) and after incubation with PECs (blue line). Variation of HSA secondary structure which is predominantly α-helix following incubation with PECs demonstrates the interaction between HSA and PECs.

SDS-page electrophoresis was performed to more accurately determine the serum protein interaction with nanoparticles. Despite of DLS results showing the thiolated nanoparticle size was significantly increased following serum incubation, the acrylamide page analysis (Figs. 2B and S3) revealed that lower amounts of proteins attached to the thiolated PECs in compare with non-thiolated ones. Hence, it was settled that the increase in size of thiolated nanoparticles following incubating in serum is probably due to the other phenomenon mentioned in sec. 2.3.1 rather than corona effect.

Furthermore, it seems that by increasing D/Ch ratio and decreasing Ch Mw, the amounts of proteins attached to the nanoparticles was diminished. It shows that dextran can effectively decreases the biointerference interactions as also affirmed by other mentioned experiments.

In fact, it seems there is less affinity between dextran and proteins in compare with chitosan. We suppose the interaction of the dextran enriched nanoparticles with proteins is less and unstable.

#### CD spectroscopy

As also shown in SDS-page analysis (Fig. 2B), one of the proteins that predominantly interacted with nanoparticles is HSA, as it is the chief protein in serum. Following evolution of protein corona by interaction of proteins with nanoparticles, secondary structure of proteins is reformed causing agglomeration of them^47^.

Herein, interaction of HSA with thiolated PEC of Ch18 with D/Ch ratio of 1 was investigated as a model via comparing the CD spectra of pure HSA and mixture of HSA and PECs. As illustrate in Fig. 2C, HSA secondary structure is mainly α-helix, which is decreased consequence of interaction with PECs.

#### LC-MS/MS spectroscopy

LC-MS/MS spectroscopy was used to evaluate protein corona composition. Fig. S4 presents the proteins interacted with the PECs of TD and Ch18 with D/Ch ratio of 0.2 (fig. S4A) and 5 (fig. S4B), and the PECs of CMD and Ch18 with D/Ch ratio of 5 (Fig. S4C). These structures were selected to evaluate the effect of thiolation and D/Ch ratio on protein corona. Despite of quantity of the attached proteins which was studied by SDS-page, protein corona identities of the various studied PECs are similar to each other mostly enriched by protein C, hemoglobin subunits and apolipoprotein AI and AII. However, some proteins individually attracted to the specific PEC, or significant difference was observed between absorption levels. For instances, protein C had more interaction with thiolated PEC, serotransferin mostly absorbed on the PEC with additional Ch content, and inter-alpha- trypsin inhibitor interaction associated to the PEC structure which interacted with the PECs with D/Ch ratio of 5 rather than D/Ch ratio of 0.2. The apolipoprotein classes and quantities were also varied among PEC protein coronas. Previous studies demonstrated that the interplay of different parameters including nanoparticle surface chemistry, incubation condition, and serum protein physicochemical properties such as protein Mw, size, electrostatic charge, and chemical composition dictate the protein corona identity^48^. The interaction of proteins with nanoparticles occurs by covalent and non-covalent bonds. Herein, the studied PECs have negative zeta potential due to the neutralizing of Ch in serum condition with pH >6.5 and negative charge of dextran. Hemoglobin possesses the positive charge in serum condition and was adsorbed to the negative charge PECs. Furthermore it could be attached to the thiolated PECs via disulfide bonds between cysteine residues^46^. Apo-lipoproteins which are amphipathic molecules could also absorb on PECs. They are small proteins with negative charge in serum and bind to the lipid particles. Saha *et al*. demonstrated that levels of lipoproteins in protein corona depends on chemistry of nanoparticle surface as it decreased on functionalized gold nanoparticles in 10% FBS medium following increasing the hydrophobicity of nanoparticle surface^41^. Protein C which is a glycoprotein also dominantly covered the PECs. The surface of particles consists of large number of carboxyl, amine, hydroxyl and thiol (in case of using TD in PEC structure) groups that involve in interactions with protein functional moieties. Although albumin has the most percentage in serum, we assume that the mentioned proteins have more affinity to the PEC functional groups.

Moreover, the interaction of the specific proteins can affect the biodistribution, clearance, and uptake of the nanoparticles by macrophages and target cells^49,50^. Apolipoproteins have critical role in cardiovascular and neurodegenerative diseases and adsorption of them on nanoparticle surface influences the nanoparticle biodistribution^41^. They also reported that apolipoproteins and complement proteins induce the uptake of nanoparticles by macrophages. However, according to our results obtained from *in vivo* study, we assume these proteins could improve the solubility of Ch based nanoparticles in serum pH, as they improve the solubility of lipid particles in blood.

Protein C identified as autoprothrombin IIA and blood coagulation factor XIV is a proenzyme that following its activation regulates anticoagulation, inflammation, cell death, and maintains the permeability of blood vessels. Although the role of some histidine rich glycoprotein (HRG) in prohibiting phagocytosis of nanoparticles with HRG enriched protein corona by macrophages was previously demonstrated^51^, the effect of protein C, which is also a glycoprotein, has remained unclear.

**Figure 3:**
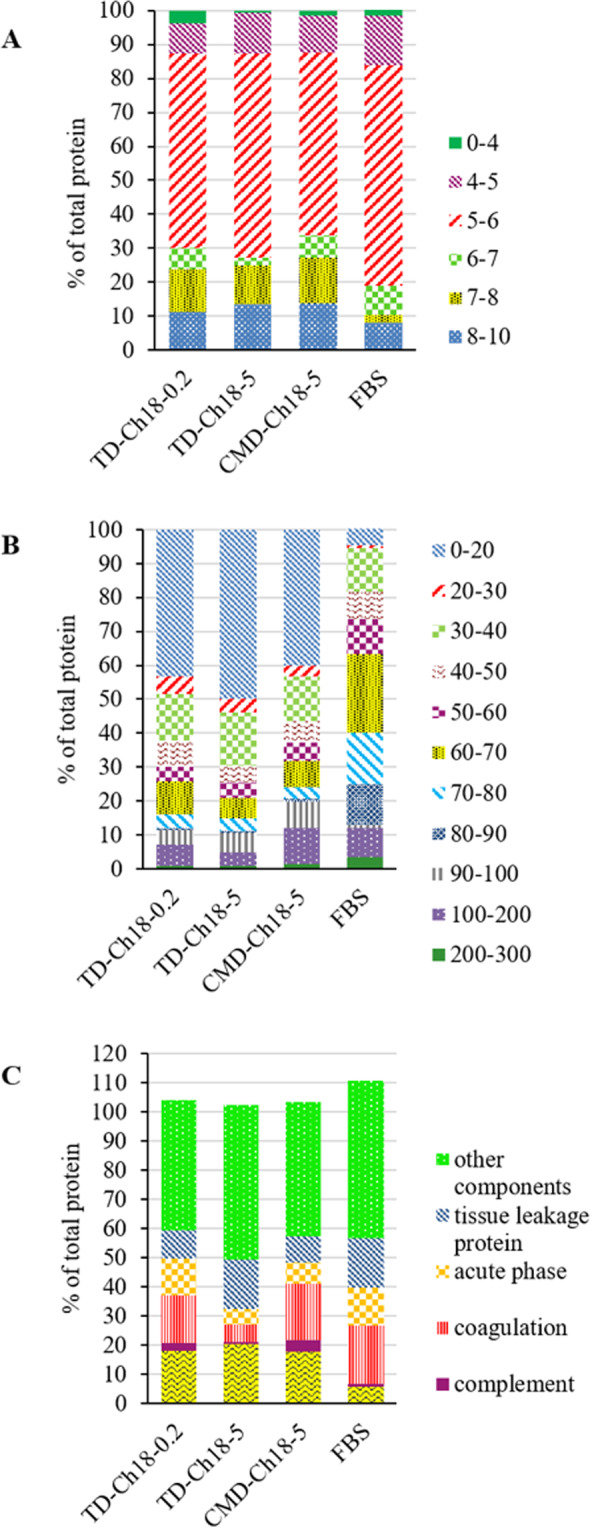
Classification of protein corona composition for the PECs consist of chitosan 18 KD (Ch18) thiolated dextran (TD) with dextran to chitosan ratio (D/Ch) of 0.2 (TD-Ch18-0.2), D/Ch ratio of 5 (TD-Ch18-5), and carboxymethyl dextran (CMD) with D/Ch ratio of 5 (CMD-Ch18-5). according to their PI (**A**) and molecular weight (**B**) and physiological function (**C**).

To better discuss the result of the test, Fig. 3 indicates the classification of hard corona composition for the PECs based on isoelectric point (pI, A), Mw (B), and physiological function (C) of the proteins.

Most of the interacted proteins, due to their high abundance in serum, have the pI between 5 to 6 and the negative charge in pH of the serum. In hard corona, the portion of proteins with neutral and positive charges considerably increased in compare with FBS contents which affirms the non-electrostatic (pI, 7-8) and electrostatic interaction (pI, 8-10) between negatively charge nanoparticles and positively charge proteins, as mentioned about interaction of hemoglobin subunits. Furthermore, the interaction of highly negative proteins with pI<4 was limited with the highly negative PECs in serum (D/Ch 5), yet it is noticeable in the case of the PECs with D/Ch 0.2 that decorated with higher number of Ch amine moieties. In fact, the interaction of Alpha-1-acid glycoprotein (AGP) with pI of 2.7 with these PECs is dominant. AGP (acute phase protein) is the plasma protein acting as drug transporters in blood system. However, we also assume that, Ch enriched PECs (D/Ch 0.2) interacted with AGP binding sites. In a study, the adsorption of plasma proteins to the different polysaccharide including dextran and Ch investigated which confirmed the extensive adsorption of AGP to Ch. Also, it was indicated that lower amounts of dextran adsorbe to the both serum albumin and AGP in compare with Ch^52^. Such binding of Ch to the glycoproteins are also observed in body mucus layer as it is well known as a mucoadhesive polymer. It was demonstrated that electrostatic, hydrogen and hydrophobic bonds have role in mucoadhesion^53^. Wan *et al*. indicated that glycosylation of protein corona significantly affects the colloidal stability and nanoparticle-cell interaction^54^. Protein Mw is another main factor determining a corona construction. As illustrates in Fig. 3B, major part of the protein corona composition consists of the proteins with Mw of <20 KD while they are not higher abundant in serum. We supposed that this phenomenon is due to the small size of nanoparticles. Hemoglobin subunits, protein C, and apolipoproteins are the abundant protein in corona which have low Mw.

Physiological classification of the proteins (Fig. 3C) demonstrates the role of corona on biodistribution and fate of PECs in different body situation including disease. Saha *et al*. showed the correlation between macrophage uptake and class of corona proteins. It seems that complement, immunoglobulin, acute phase and lipoproteins mostly influence the phagocytosis^41^. Herein, lipoproteins had higher abundance in protein corona composition of the PECs in compare with FBS.

### Cellular *in vitro* study

#### Effects of serum and pH on uptake of nanoparticles

To find the correlation between structural parameters, medium condition, protein corona, and cellular uptake of PECs, this set of PECs was selected for *in vitro* tests to simultaneously evaluate effects of D/Ch ratio, thiolation of dextran, Ch Mw, PEC size, and their zeta potential on cellular uptake of the PECs. Furthermore, effects of FBS and pH of the medium on uptake of the PECs were studied.

**Figure 4:**
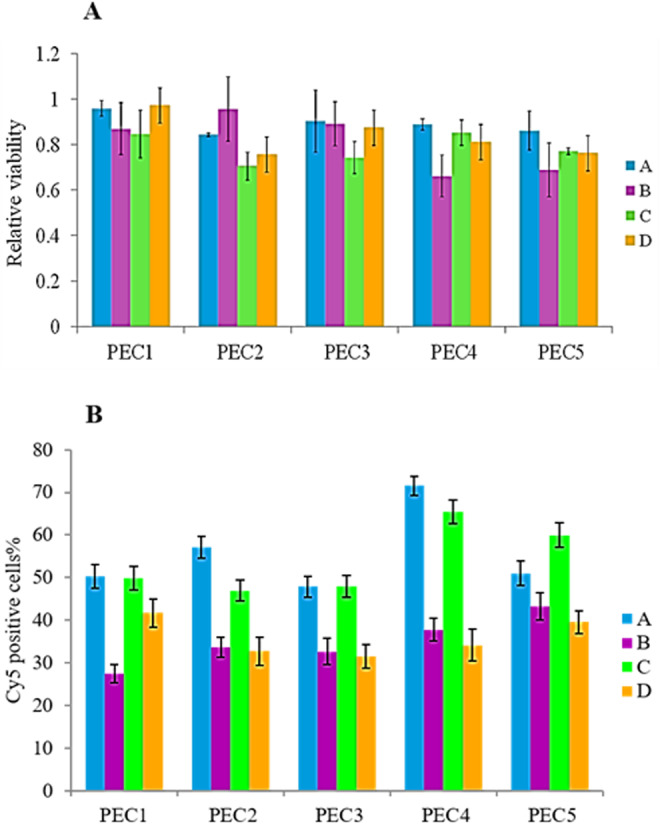
*In vitro* tests; (**A**) Flow cytometery analysis to detect the percentage of Cy5 positive cells after transfection by various PECs in different condition. (**B**) The proliferation of MCF-7 incubated with the PECs in various culture mediums. The PEC composed of Ch18 and TD with D/Ch ratio of 0.2 (PEC1), the PEC composed of Ch18 and CMD with D/Ch ratio of 5 (PEC2), the PEC composed of Ch9 and TD with D/Ch ratio of 5 (PEC3), the PEC composed of Ch18 and TD with D/Ch ratio of 5 (PEC4), the chitosan polyplexes composed of Ch18 and Cy-5 oligonucleotide (PEC5). DMEM + FBS 10% with pH 7.4 (**A**), DMEM with pH 7.4 (**B**), DMEM + FBS 10% with pH 6.8 (**C**), DMEM with pH 6.8 (**D**).

As shown in Fig. 4A, we found out that uptake of the PECs was noticeably enhanced by FBS (p < 0.05). However, it was not significant for the thiolated PEC composed of Ch18 with D/Ch ratio of 0.2 in acidic medium, and Ch-DNA complex in the medium with pH, 7.4. Previous researches indicated that serum proteins influence uptake of nanoparticles by cells as the structurally same nanoparticles can lead to very different biological outcomes in the presence or absence of protein corona^55^. Herein, different parameters have reverse effects on the test results. First, FBS lead to an increase in uptake of the PECs because cell function rises by addition of serum. Second, interaction of nanoparticles with serum proteins leads to an increase in size of the nanoparticles and changes their surface properties such as zeta potential, consequently, it decreases PEC uptake. Third, existence of some proteins in corona such as inter alpha-trypsin inhibitor chains and lipoproteins could enhance the cellular uptake^56,57^. It seems that none of these events was dominant for the PECs with D/Ch ratio of 0.2 and Ch-DNA complex while the interaction of all mentioned factors play role. The other PECs had small interaction with serum proteins and very low variation in their size after addition of serum. In fact, they only benefit from positive aspects of serum and protein corona.

Despite of FBS, pH effect was insignificant factor in PEC uptake excluding thiolated PEC composed of Ch18 with D/Ch ratio of 0.2 in FBS free medium that the number of the fluorescent positive cells was significantly higher at acidic pH due to the more positive charges on the surface of the PECs which interact with the cell membrane phosphate groups. Also, it seems that Ch-DNA complex uptake is slightly higher at acidic pH in FBS contained medium. Hoemann *et al*. verify that uptake of Ch is affected by soluble versus microparticle state, thus it is enhanced by serum-induced cell metabolism and lactate-based medium acidification in presence of 10% FBS^58^. Furthermore, uptake of Ch nanoparticles is noticeably enhanced in acidic medium because of Ch amine group ionization and positive zeta potential of the complexes.

Since the zeta potential of the PECs with D/Ch ratio of 5 is negative, even in acidic medium, pH did not have meaningful effect.

To investigate the effect of thiolation, thiolated and non-thiolated PECs of Ch18 with D/Ch ratio of 5 were compared. Many studies indicated the priority of thiolation in oral delivery systems for increasing mucoadhesion, residence time in site of action, and cellular uptake^59^ but MCF 7 is a breast cancer cell line and does not have mucus layer, hence the significant difference was unforeseen. It was no significant difference between thiolated and non-thiolated PECs in FBS free medium but the number of the fluorescent positive cells was meaningfully higher after incubation with thiolated PEC in presence of FBS (p < 0.05). The stability of thiolated PECs was higher than non-thiolated PECs due to disulfide cross linking in thiolated PEC structure which improve the Cy5-oligo preservation in serum contained medium. Furthermore, protein corona of the PECs affected the cellular uptake. As shown by SDS-page electrophoresis evolution of protein corona for thiolated PECs was less than non-thiolated ones.

The effect of Ch Mw was studied by the comparison among thiolated PECs with D/Ch ratio of 5. Same as thiolation effect, only in FBS contained medium the significant difference was observed between the samples, and the PEC consisted of Ch18 had higher uptake by the cells. Based on our previous study, the stability of the PECs with larger Ch is higher in FBS^35,43^.

By the comparison between the thiolated PECs consisted of Ch18 with different D/Ch ratio, it was found that higher D/Ch ratio led to the more number of the fluorescent positive cells in FBS contained medium, possibly due to the smaller size in serum condition and lower interaction with proteins.

In acidic FBS free condition, the PEC with D/Ch ratio of 0.2 had the most internalization to the cells in compare with the other nanoparticles; but in neutral FBS free condition, the PEC with D/Ch ratio of 5 had the most. Nevertheless, in FBS free medium, these differences were not statistically significant. Obviously, there was an interaction between effects of the parameters. For example, no significant difference was observed between the PECs with various D/Ch ratios when Ch Mw or dextran thiolation was also varied, and how the structural parameters influenced the uptake depended on incubation condition.

A significant uptake of the thiolated PECs with D/Ch ratio of 5 was also confirmed by confocal microscopy. As shown in Fig. S5, the thiolated PECs noticeably transfected MCF-7 cells. It asserts that these nanoparticles are the appropriate systems for effective gene and drug delivery. We also advocate the thiolated PECs for transfection of colon cells due to the presence of thiol moieties, which enhance the uptake of nanoparticles by the mucoadhesion mechanism.

#### Cell toxicity of the PECs in various culture mediums

To demonstrate the relation between PEC structural factors, medium condition, protein corona and biocompatibility of the PECs, cell toxicity of different formulation on MCF7 cell line was investigated using MTT assay. Since pH and serum quantity affect cell proliferation, to estimate their influence on toxicity of the PECs with various structures, we consider a separate negative control for each test which were the cells incubated in similar medium without PECs. Figure 4B illustrates the cytotoxicity of the PECs. As the toxicity of the PECs is negligible, serum and pH only have a negligible effect on the results of the test. Ch nanoparticles are mostly toxic except in serum free medium with pH 7.4. It seems that adding dextran, especially TD, increased the biocompatibility of PECs. The PEC composed of Ch18 and TD with D/Ch 0.2 had no toxicity in compare with control in all culture medium; however, by rising D/Ch the toxicity of the PECs slightly raised.

### *In vivo* biodistribution tests

#### Preparation and characterization of radio-labeled PECs

^68^Ga is a positron-emitting isotope generated from ^68^Ge in ^68^Ge/^68^Ga generators and was used in diagnostic PET scans. The ^68^Ga labeled Ch-DOTA were obtained by conjugating Ch to DOTA chelating agent and incubating with ^68^Ga. After purification, the radiolabeled Ch-DOTA was complexed with Ch and CMD/TD to obtain ^68^Ga labeled nanoparticles.

According to the TLC outcomes, ^68^Ga citrate complexes migrated to R_f_ 0.6 while the radiolabeled Ch-DOTA retained at R_f_ 0.1, and the yield of labeling procedure was 50–60%, approximately. Following purification of the radiolabeled Ch-DOTA, the unbounded ^68^Ga was decreased to less than 20%. Because of the short half-life of ^68^Ga, 68 min, it is not possible to repeat the purification procedure to further separate the unbounded ^68^Ga. The stability of radio-labeled PECs in serum was investigated. TLC shows the radiolabeled PECs are stable in serum condition and it was found that the release of ^68^Ga was less than 10% after 3 hours (Data is not shown).

#### PET-CT scan

**Figure 5:**
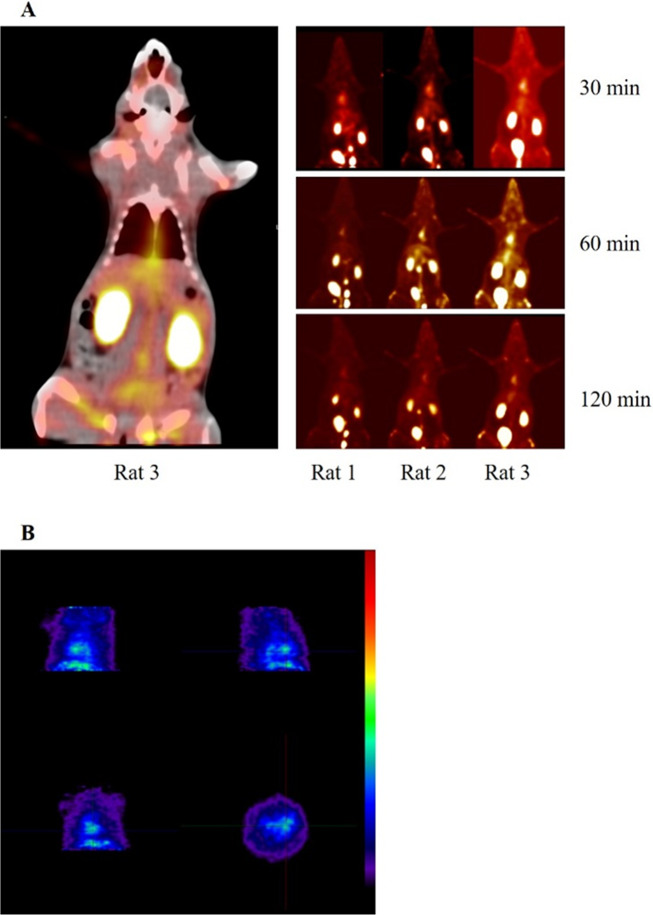
*In vivo* study; (**A**) PET/CT scan of the rat models treated by tail vein injection of the ^68^Ga-labeled nano PECs. The scan was accomplished in three time intervals of 30, 60, 120 min. Bidistribution of the nano PECs consists of Ch18 and CMD with D/Ch ratios of 5 (rat 1 as a sample of group1), Ch18 and TD with D/Ch ratios of 0.2 (rat 2 as a sample of group 2) and 5 (rat 3 as a sample of group 3) was investigated. (**B**) Blood pool and cardiac uptake of the PEC composed of TD and Ch18 with D/Ch ratio of 5 determined by animal PET scan of the rat model heart.

It seems that the PEC composed of Ch18 and TD with D/Ch ratio of 5 is the best formulation which had the most cellular uptake in FBS contained culture medium. Moreover, the size of this PEC is appropriate for *in vivo* tests. The proper hydrodynamic diameter seems to be between 10-100 nm since the particles smaller than 10 nm are quickly cleared by kidney or through extravasation, and the particles larger than 100 nm are cleared through opsonization and reticuloendothelial system. Furthermore, efficacy of neutral nanoparticles (-10 to +10 mv) tend to be higher^60^. As shown in Table 3, the PECs with D/Ch ratio of 0.2 are smaller than 100 nm, yet the size extremely changed after incubation with FBS by protein corona formation, swelling or other physical phenomenon. However, since the method of nanoparticle preparation for size determination following evolution of corona could increase the aggregation of nano PECs, we could not make the decision based on the results of the test. Thus, *in vivo* bidistribution of the nanoparticles including the PECs composed of Ch18 and CMD with D/Ch ratios of 5 (group 1), Ch18 and TD with D/Ch ratios of 0.2 (group 2) and 5 (group 3) was investigated (Fig. 5).

PET/CT images of 3 groups of rat models clearly show the activity concentration in the kidneys and bladder due to incidence of very small nanoparticles in formulation that have glomerular filtration. Although the purification process was accomplished, presence of the unbounded ^68^Ga in formulations is probable that has renal clearance. Also, there was a significant uptake in the heart of the 3 groups of rat models that was due to the accumulation of the nanoparticles in tissue and blood pool. The animal PET scan was conducted to accurately determine the biodistribution and evaluation of blood pool or heart tissue uptake of the nanoparticles. It indicated that very small portion of the nanoparticles accumulated into heart tissue which is associated to the protein corona composition of the nanoparticles that was enriched by lipoproteins. Surprisingly, there is no meaningful uptake in the lung site in the representative rat models which indicates the appropriate size distribution of the nanoparticles in serum as the large nanoparticles mostly accumulate into the lung tissue. There was low uptake in spleen and liver in comparison with the other mentioned tissues that demonstrates low opsonization and clearance by Kupffer cells and long blood circulation time of the nanoparticles.

For quantitative assessment of target uptake behavior, maximum and mean activity concentrations (Bq/cc) were measured in all PET imaging studies (Table S1). It seems that less than 10% of the nanoparticles had liver and spleen uptake while cardiac to liver uptake ratio was between 1.25 to 2.97 depends on formulations and it changed over the time. In group 1, 20% increase in liver activity concentration was observed during 30 min to 1 hour after injection due to the opsonization of the nanoparticles by reticule-endothelial systems; yet after 2 hours, the detected liver activity was decreased about 75%, perhaps by entrohepatic exertion of nanoparticles. Similar phenomenon was also observed in two other rat groups. In group 2, 34% increase in liver activity concentration was found in first hour of injection and then it was reduced to 1/3. In group 3, two times increase in liver activity concentration was observed in first 1 hour followed by decrease to half after 2 hours. We assume that the fast entrohepatic exertion of nanoparticles could be due to the nature of the nanoparticles that composed of low Mw biodegradable polymers.

About heart localization of the nanoparticles, there was no significant difference between 3 groups. Since the nanoparticles were covered by protein corona enriched by lipoproteins, thus the high cardiac uptake was expectable. Similar to the liver uptake, cardiac uptake was increased about 30% during first hour followed by decreasing to 1/4 after 2 hours, in rat models of group 1 and 2. Also, 4 times increase in activity concentration was observed in rat models of group 3 heart after 1 hour, but it was diminished 3.5 times after 2 hours. We supposed that the decrease of activity in heart blood pool during second hours post administration was because of tissue uptake of nanoparticles.

By considering low liver and lung uptake of the PECs, we supposed that the *in vivo* size of nanoparticles in serum was not high as demonstrated by *in vitro* test using DLS. It seems that, centrifuging of the nano PECs could increase the rate of irreversible aggregation. Hence, more trusting prediction is driven from SDS-page electrophoresis and LC-MS/MS spectroscopy. However, the recent studies indicated that it was not always the significant correlation between *in vitro* and *in vivo* corona due to the wide variety of molecular species in the *in vivo* corona, dynamics of blood flow, interaction with circulating and endothelial lining cells, and immune responses. Unfortunately, the *in vivo* corona evaluation is challenging because of the insufficient recovery of the nanoparticles following administration^61–63^.

Although the *in vitro* and *ex-vivo* corona studies outcomes is uncertain, it could be useful for some initial predications. In this study, we could find an agreement between quantity and composition of PEC protein corona and nanoparticle biodistribution based on LC-MS/MS spectroscopy and SDS-page electrophoresis results and initial size of our nanoparticles without corona.

## Conclusion

Protein corona influences biodistribution and cell internalization of nanoparticles. To improve the biodistribution and enhance the circulation time of chitosan based nanoparticles, the biointerference of the PECs of chitosan and dextran were thoroughly investigated. Chitosan and CMD/TD PECs have thin protein corona layer, in compare with inorganic nanoparticles, mostly consists of lipoproteins, protein C, and hemoglobin which could affect the biodistribution and cellular uptake of nanoparticles. The nanoparticles possess the long circulation time with low liver uptake due to their suitable size distribution in serum. The heart tissue uptake of the PECs was minor and could be due to the lipoprotein enriched corona structure. By considering the obtained results beside biocompatibility and safety of the chitosan and dextran, we conclude that the presented modified nanoparticles could be suggested as prosperous gene and drug delivery systems.

## Supplementary information

supporting information (revised).

## References

[1] Caracciolo G (2010). Surface adsorption of protein corona controls the cell internalization mechanism of DC-Chol–DOPE/DNA lipoplexes in serum. Biochimica et Biophysica Acta (BBA)-Biomembranes.

[2] Safi M, Courtois J, Seigneuret M, Conjeaud H, Berret J-F (2011). The effects of aggregation and protein corona on the cellular internalization of iron oxide nanoparticles. Biomaterials..

[3] Zhang H (2018). Ligand Size and Conformation Affect the Behavior of Nanoparticles Coated with *in Vitro* and *in Vivo* Protein Corona. ACS Applied Materials & Interfaces.

[4] Cui M (2014). Quantitative study of protein coronas on gold nanoparticles with different surface modifications. Nano Research..

[5] Owens DE, Peppas NA (2006). Opsonization, biodistribution, and pharmacokinetics of polymeric nanoparticles. International journal of pharmaceutics.

[6] Aggarwal P, Hall JB, McLeland CB, Dobrovolskaia MA, McNeil SE (2009). Nanoparticle interaction with plasma proteins as it relates to particle biodistribution, biocompatibility and therapeutic efficacy. Advanced drug delivery reviews.

[7] Lundqvist M (2008). Nanoparticle size and surface properties determine the protein corona with possible implications for biological impacts. Proceedings of the National Academy of Sciences.

[8] Laurent S, Burtea C, Thirifays C, Rezaee F, Mahmoudi M (2013). Significance of cell “observer” and protein source in nanobiosciences. Journal of colloid and interface science.

[9] Yu M, Zhou C, Liu J, Hankins JD, Zheng J (2011). Luminescent gold nanoparticles with pH-dependent membrane adsorption. Journal of the American Chemical Society.

[10] Tenzer S (2013). Rapid formation of plasma protein corona critically affects nanoparticle pathophysiology. Nature nanotechnology..

[11] Casals E, Pfaller T, Duschl A, Oostingh GJ, Puntes V (2010). Time evolution of the nanoparticle protein corona. ACS nano..

[12] Albanese A, Tang PS, Chan WC (2012). The effect of nanoparticle size, shape, and surface chemistry on biological systems. Annual review of biomedical engineering.

[13] Wolfram J (2014). The nano-plasma interface: implications of the protein corona. Colloids and Surfaces B: Biointerfaces.

[14] Sacchetti C (2013). Surface polyethylene glycol conformation influences the protein corona of polyethylene glycol-modified single-walled carbon nanotubes: potential implications on biological performance. ACS nano..

[15] Runa, S., Hill, A., Cochran, V. L., Payne, C. K. editors. PEGylated nanoparticles: protein corona and secondary structure. SPIE NanoScience+ Engineering; 2014: International Society for Optics and Photonics.

[16] Pelaz, B. *et al*. Surface Functionalization of Nanoparticles with Polyethylene Glycol (PEG): Effects on Protein Adsorption and Cellular Uptake. *ACS nano*. (2015). 10.1021/acsnano.5b01326 26079146

[17] Sánchez-Moreno P (2015). Balancing the effect of corona on therapeutic efficacy and macrophage uptake of lipid nanocapsules. Biomaterials..

[18] Moore A, Marecos E, Bogdanov A, Weissleder R (2000). Tumoral distribution of long-circulating dextran-coated iron oxide nanoparticles in a rodent model 1. Radiology..

[19] Peng, M. *et al*. Dextran-coated superparamagnetic nanoparticles as potential cancer drug carriers *in vivo*. *Nanoscale* (2015). 10.1039/c5nr01382h 26062012

[20] Gref R (2000). ‘Stealth’corona-core nanoparticles surface modified by polyethylene glycol (PEG): influences of the corona (PEG chain length and surface density) and of the core composition on phagocytic uptake and plasma protein adsorption. Colloids and Surfaces B: Biointerfaces.

[21] Natte K (2013). Impact of polymer shell on the formation and time evolution of nanoparticle–protein corona. Colloids and Surfaces B: Biointerfaces.

[22] Guo H (2015). Theranostic magnetoliposomes coated by carboxymethyl dextran with controlled release by low-frequency alternating magnetic field. Carbohydrate Polymers..

[23] McBain SC, Yiu HHP, Dobson J (2008). Magnetic nanoparticles for gene and drug delivery. International Journal of Nanomedicine.

[24] Anitha A (2011). Preparation, characterization, *in vitro* drug release and biological studies of curcumin loaded dextran sulphate–chitosan nanoparticles. Carbohydrate Polymers..

[25] Delair T (2011). Colloidal polyelectrolyte complexes of chitosan and dextran sulfate towards versatile nanocarriers of bioactive molecules. European Journal of Pharmaceutics and Biopharmaceutics.

[26] Lin Y-S, Okamoto Y, Minami S (2010). Effects of chitosan-carboxymethyl dextran nanoparticles on cell proliferation and on serum cytokine regulation. Journal of biomedical nanotechnology.

[27] Lin Y-S (2009). Preparation of stable chitosan-carboxymethyl dextran nanoparticles. Journal of nanoscience and nanotechnology.

[28] Tiyaboonchai W (2013). Chitosan nanoparticles: a promising system for drug delivery. Naresuan University. Journal: Science and Technology.

[29] Mao S, Sun W, Kissel T (2010). Chitosan-based formulations for delivery of DNA and siRNA. Advanced drug delivery reviews.

[30] Parveen S, Sahoo SK (2011). Long circulating chitosan/PEG blended PLGA nanoparticle for tumor drug delivery. European Journal of Pharmacology.

[31] Li X (2012). Interaction of bovine serum albumin with self-assembled nanoparticles of 6-O-cholesterol modified chitosan. Colloids and Surfaces B: Biointerfaces.

[32] Mao H-Q (2001). Chitosan-DNA nanoparticles as gene carriers: synthesis, characterization and transfection efficiency. Journal of Controlled Release.

[33] Lin YS (2012). Characterization of Chitosan-Carboxymethyl Dextran Nanoparticles as a Drug Carrier and as a Stimulator of Mouse Splenocytes. Journal of Biomaterials Science, Polymer Edition.

[34] Tekie FSM (2017). Nano polyelectrolyte complexes of carboxymethyl dextran and chitosan to improve chitosan-mediated delivery of miR-145. Carbohydrate Polymers..

[35] Kiani M (2016). Thiolated carboxymethyl dextran as a nanocarrier for colon delivery of hSET1 antisense: *In vitro* stability and efficiency study. Materials Science and Engineering: C..

[36] Dinarvand M (2015). Oral delivery of nanoparticles containing anticancer SN38 and hSET1 antisense for dual therapy of colon cancer. International journal of biological macromolecules.

[37] Almalik A (2017). Hyaluronic Acid Coated Chitosan Nanoparticles Reduced the Immunogenicity of the Formed Protein Corona. Scientific reports.

[38] Shahnaz G, Perera G, Sakloetsakun D, Rahmat D (2010). Bernkop-Schnürch A. Synthesis, characterization, mucoadhesion and biocompatibility of thiolated carboxymethyl dextran–cysteine conjugate. Journal of Controlled Release.

[39] Lundqvist M (2011). The evolution of the protein corona around nanoparticles: a test study. ACS nano..

[40] Cedervall T (2007). Detailed identification of plasma proteins adsorbed on copolymer nanoparticles. Angewandte Chemie International Edition.

[41] Saha K (2016). Regulation of macrophage recognition through the interplay of nanoparticle surface functionality and protein corona. ACS nano..

[42] Mahmoudi M (2013). Temperature: the “ignored” factor at the nanobio interface. ACS nano..

[43] Tekie FSM (2015). Chitosan polyplex nanoparticle vector for miR-145 expression in MCF-7: Optimization by design of experiment. International journal of biological macromolecules.

[44] Nel AE (2009). Understanding biophysicochemical interactions at the nano-bio interface. Nature materials..

[45] Bigucci F (2008). Chitosan/pectin polyelectrolyte complexes: Selection of suitable preparative conditions for colon-specific delivery of vancomycin. European Journal of Pharmaceutical Sciences.

[46] Moerz ST, Kraegeloh A, Chanana M, Kraus T (2015). Formation Mechanism for Stable Hybrid Clusters of Proteins and Nanoparticles. ACS Nano..

[47] Gebauer JS (2012). Impact of the Nanoparticle–Protein Corona on Colloidal Stability and Protein Structure. Langmuir..

[48] Del Pino P (2014). Protein corona formation around nanoparticles–from the past to the future. Materials Horizons.

[49] Corbo, C., Mahmoudi, M., Farokhzad, O. C. Personalized cancer-specific protein corona affects the therapeutic impact of nanoparticles. *AACR* (2018).

[50] Caracciolo G, Farokhzad OC, Mahmoudi M (2017). Biological Identity of Nanoparticles *In Vivo*: Clinical Implications of the Protein Corona. Trends in Biotechnology.

[51] Fedeli C (2015). The functional dissection of the plasma corona of SiO_2_-NPs spots histidine rich glycoprotein as a major player able to hamper nanoparticle capture by macrophages. Nanoscale..

[52] Isobe T (2016). Adsorption of histones on natural polysaccharides: The potential as agent for multiple organ failure in sepsis. International Journal of Biological Macromolecules.

[53] Sogias IA, Williams AC, Khutoryanskiy VV (2008). Why is Chitosan Mucoadhesive?. Biomacromolecules..

[54] Wan S (2015). The “sweet” side of the protein corona: effects of glycosylation on nanoparticle–cell interactions. ACS nano..

[55] Lesniak A (2012). Effects of the Presence or Absence of a Protein Corona on Silica Nanoparticle Uptake and Impact on Cells. ACS nano..

[56] Ritz S (2015). Protein Corona of Nanoparticles: Distinct Proteins Regulate the Cellular Uptake. Biomacromolecules..

[57] Safavi-Sohi R (2016). Bypassing protein corona issue on active targeting: zwitterionic coatings dictate specific interactions of targeting moieties and cell receptors. ACS applied materials & interfaces.

[58] Hoemann CD (2013). Chitosan rate of uptake in HEK293 cells is influenced by soluble versus microparticle state and enhanced by serum-induced cell metabolism and lactate-based media acidification. Molecules..

[59] Bernkop-Schnürch A (2005). Thiomers: a new generation of mucoadhesive polymers. Advanced drug delivery reviews.

[60] Wilhelm S (2016). Analysis of nanoparticle delivery to tumours. Nature Reviews. Materials..

[61] Hadjidemetriou M, Kostarelos K (2017). Nanomedicine: evolution of the nanoparticle corona. Nature nanotechnology..

[62] Müller LK (2018). The Transferability from Animal Models to Humans: Challenges Regarding Aggregation and Protein Corona Formation of Nanoparticles. Biomacromolecules..

[63] Stepien G (2018). Effect of Surface Chemistry and Associated Protein Corona on the Long-Term Biodegradation of Iron Oxide Nanoparticles *In Vivo*. ACS Applied Materials & Interfaces.

