# Supplementary material for: Controlling evolution of protein corona: a prosperous approach to improve chitosan-based nanoparticle biodistribution and half-life
Source: Sci Rep. 2020 Jun 15;10:9664. doi: 10.1038/s41598-020-66572-y (PMC7295777; doi:10.1038/s41598-020-66572-y)
Supplement: Supplementary file 1 — supporting information (revised). [file 41598_2020_66572_MOESM1_ESM.docx]

**Supporting Informations**

**Controlling evolution of protein corona: a prosperous approach to improve chitosan-based nanoparticle biodistribution and half-life**

Farnaz Sadat Mirzazadeh Tekie^a^, Maliheh Hajiramezanali^b^, Parham Geramifar^c^, Mohammad Raoufi^d^, Rassoul Dinarvand^a,e^, Masoud Soleimani^f,g^, Fatemeh Atyabi^a,e,*^

^a^Nanotechnology Research Centre, Faculty of Pharmacy, Tehran University of Medical Sciences, Tehran, Iran

^b^Department of Radipharmacy, Faculty of pharmacy, Tehran University of Medical Sciences, Tehran, Iran

^c^Research Center for Nuclear Medicine, Shariati Hospital, Tehran University of Medical Sciences, Tehran, Iran

^d^Department of Pharmaceutical Nanotechnology, Faculty of pharmacy, Tehran university of medical sciences, Tehran, Iran

^e^Department of Pharmaceutics, Faculty of Pharmacy, Tehran University of Medical Sciences, P.O. Box 14155-6451, Tehran, Iran

^f^Department of Molecular Biology and Genetic Engineering, Stem Cell Technology Research Center, P.O. Box 14155-3174, Tehran, Iran

^g^Department of Hematology, School of Medical Sciences, Tarbiat Modares University, P.O. Box 14115-111, Tehran, Iran

∗Corresponding author at: Faculty of Pharmacy, Tehran University of Medical Sciences, Nanomedicine, Enghelab St., Teheran, Iran. Tel.: +98 21 66959052; fax: +98 21 66959052. E-mail address: atyabifa@tums.ac.ir (F. Atyabi).

**Materials and Methods**

1. **Materials**

CMD (10-20 KD), L-cysteine hydrochloride monohydrate (L-Cys), 5,5′-dithiobis-(2-nitrobenzoic acid) (DTNB), ethidium bromide (EtBr), ethylenediaminetetraacetic acid (EDTA), N-(3-Dimethylaminopropyl)-N′-ethylcarbodiimide hydrochloride (EDC), and trypsin were obtained from Sigma-Aldrich (Missouri, USA). Ch (MW: 400 KD, degree of deacetylation: 87%) was from Primex (Siglufjörður , Norway). Heat inactivated fetal bovine serum (FBS), Dulbecco’s modified Eagle’s medium (DMEM), and 3-(4,5-Dimethylthiazol-2-yl)-2,5-Diphenyltetrazolium Bromide (MTT) were purchased from GIBCO (Life Technologies Inc., New York, USA). Cy5 conjugated scrambled DNA (5’- CATCGAAATCGTTGCAGTTAC-3’) were provided from Macrogene (Seoul, Korea). S-2-(4-Isothiocyanatobenzyl)-1,4,7,10-tetraazacyclododecane tetraacetic acid (p-SCN-Bn-DOTA) was purchased from Macrocyclics Inc. (Texas, USA). A ^68^Ge/^68^Ga generator (20 mCi/elution) was obtained from Pars Isotope Co. (Karaj, Iran). Other analytical grade reagents were from Merck (Darmstadt, Germany).

1. **Synthesis and purification of TD**

The solution consists of CMD (60 g/l) and EDC (150 mM) was stirred for about 45 min at room temperature. Following addition of L-Cys (60g/l), the reaction was done under nitrogen blanket for 4 h. TD was purified using dialysis membrane (MWCO: 8KD) as described before [35].

1. **Low Mw Ch preparation and characterization**

Ch with Mw of 9 (Ch9) and 18 KD (Ch18) were obtained by nitrous acid hydrolysis of fully de-N-acetylated Ch 400 KD using 7.5 and 3.75 mg/ml of NaNO2, respectively. The average Mw of the polymers were estimated by viscometric method on the basis of intrinsic viscosity [η], as described previously [34].

1. **Thermal gravimetric analysis (TGA)**

To confirm PEC development and evaluate thermal stability of the PEC, TGA was executed on CMD, Ch, and the PECs utilizing a TGA/DTA BAHR: STA 503 thermal analyzer instrument from 25 to 600 ◦C at a heating rate of 10 ◦C/min under air.

1. **Transmission electron microscopy (TEM)**

To observe the topography and structural shape of PECs, TEM was accomplished using CEM 902A microscope (Zeiss, Germany). For this purpose, a suspension of nanoparticles in distilled water was placed on a film-coated copper grid and dried at room temperature prior to observation.

1. **Evaluating size distribution and zeta potential of the PECs**

Hydrodynamic size and polydispersity index (PDI) of the PECs were evaluated via dynamic light scattering (DLS) using Malvern Zetasizer Nano-ZS (Malvern Inst. Ltd. Malvern, UK) with a detection angle of 90° at a wavelength of 633 nm and 25°C.

Zeta potential of PECs was determined by Laser Doppler Electrophoresis using Zetasizer (Nano-ZS, Malvern, UK). The test was replicated following incubating the PECs with FBS 20% and washing by PBS.

1. **Liquid chromatography –Mass/Mass (LC-MS/Ms) spectroscopy**

LC-Ms/Ms was performed to determine protein corona construction, spectral count of peptides (SPC) and relative quantity of each protein emerged on PEC corona. The PEC-bound proteins were digested using sequencing grade modified trypsin (Promega) followed by incubating at 37 °C overnight. The LC-MS/MS spectrometry on digested PEC-bound proteins was executed utilizing an Ultimate nanoRSLC-HPLC system (Dionex) with a nano C18 RP column linked to Orbitrap Velos Pro mass spectrometer (ThermoScientific) via a Proxeon nanospray according to the previously reported method. Water/formic acid (0.05%) was served as a solvent at the flow rate of 6 µL/min to concentrate and desalt the peptides by the hydrophobic column. LC was accomplished with gradient of solvents including water/formic acid (0.05%) and acetonitrile (80%)/formic acid (0.045%) at the flow rate of 300 nL/min. The second solvent was 4% for five minutes, afterwards it increased linearly to 45% in 30 minutes and then to 95% in 5 minutes. The eluent was sprayed in the direction of the mass spectrometer capillary by a nanoemitter (Proxeon, Denmark) with the potential of 2300 V. Precursor ion masses were determined using a survey scan at a resolution of 60000 within the Orbitrap mass analyzer. The combination of the survey scan with three data dependent MS/MS scans by 30 s dynamic exclusion via CID with the linear ion-trap and HCD joint to Orbitrap detection with a resolution of 7500 was used to collect and analysis the Data by Proteome Discoverer (ThermoScientific) with SEQUEST and MASCOT (version 2.2; Matrix science) search engines using SwissProt or NCBI databases [44, 63].

1. **Circular dichroism (CD) spectroscopy**

It was explained in supporting information, S8. CD spectroscopy was performed to determine alteration of HSA protein secondary structure following interaction with the PECs. The PEC was incubated with HSA solution with the concentration of 2 mg/ml for 1 hr at 37^o^C. The HSA solution with concentration of 2 mg/ml was prepared as a control. The measurements were accomplished by means of AVIV 62 A DS CD spectrometer with a slit width of 1 μm and the scanning step size of 0.1 nm in a 1 mm path length Suprasil quarz cuvette.

1. **The nanoparticle uptake study**

MCF-7 cells were seeded a night before the test with DMEM medium containing 10% (v/v) FBS in 12-well plate with confluency of 80%. The cells were then incubated with 1ml mixtures (1:1) of the Cy5-oligo nanoparticles and culture medium at 37^o^C in 5% CO2 atmosphere (Table 3). The final concentrations of Cy5-oligo were 1 nM in the culture medium. After 1 h, the growth medium was removed and the cells were detached by trypsin-EDTA. Following quenching the enzyme by FBS and separating the cells by centrifuging, they were washed doubling time by adding PBS, centrifuging, and discarding supernatant. The cells were dispersed in 100 µl of PBS, and a percentage of florescent positive cells was measured on a flow cytometer (FACS Calibur Becton, Dickinson, United States), and analyzed by WinMidi software (Scripps Research Institute; San Diego, United States).

1. **Confocal microscopy**

The cells were seeded with 80% confluency on a cover slip put in a 6-well plate followed by incubation overnight. 1 ml of the mixture (1:1) of Cy5-oligo PECs and culture medium (test 8, table 3) was poured into each well, and incubated for 3 hr. The final concentration of Cy5-oligo was 10 nM in each well. The cells were then washed by PBS, fixed by formaldehyde, and visualized using a Nikon confocal laser microscope A1 (Nikon Inc., USA) equipped with a A1 scan head and a standard detector using a red diode laser (Melles Griot, USA) at 638 nm and utilizing a CY5 filter.

1. **Cell toxicity of the nanoparticles**

MTT assay was performed to evaluate toxicity of the nanoparticles in the various medium on MCF-7. The cells were seeded in 24-well plates (40,000 cells/well) and incubated overnight. 500 µl of the prepared mixtures of nanoparticles and the culture mediums (Table 3) with a volume ratio of 1:1 was added to each well, and the cells were incubated for 6 h. The medium was removed and the cells were washed by PBS and incubated with DMEM containing 10% of FBS for 24 h. MTT test was accomplished, and the cell viability in each situation was estimated in compare with the control, the cells which were incubated with the nanoparticle free mediums.

1. **Positron emission tomography/ computed tomography (PET/CT) study**

In vivo study was performed to compare the biodistribution of the PECs composed of Ch and CMD with D/Ch ratio of 5, Ch and TD with D/Ch ratio of 0.2 and 5. The animal experiments were carried out by considering the approved guidelines of ethical care and use of research animal at Tehran University of medical sciences. Three groups (5 rats in each groups) of male rats (obtained from animal lab in Tehran university of medical science, school of pharmacy) under ketamine-xylazine anesthesia were tail-vein injected with adequate volume of radio labeled nano PECs contained 8.5 MBq activity. Imaging properties and biodistribution of nanoparticles were assessed non-invasively with PET/CT imaging using Biograph 6 Siemens clinical scanner (Siemens AG, Erlangen, Germany). The rats were placed in supine position and CT scans performed for anatomical reference and attenuation correction (spatial resolution 1.25 mm, 80 kV, 50 mAs).

PET acquisition was performed in 3 sets of emission images starting 30, 60 and 120 min post injection. The decay corrected acquision protocol was set to 10 minutes per bed position.

PET images were reconstructed using TrueX algorithm with attenuation correction. The reconstruction settings were 2 iterations and 21 subsets to a 336×336 matrix, with a post filtering of 5 mm. Transmission data were reconstructed into a matrix of equal size by means of filtered back-projection, yielding a co-registered image set. The reconstructed PET images were then fused with CT images.

**Results and Discussion**

1. **Polymer preparation and characterization**

Ch is the preeminent positive polymer have been widely used in drug and gene delivery systems [29, 38]. To determine the effect of Ch molecular weight (Mw) on corona evolution, Ch was depolymerized effectively to the intended different Mw, 9 and 18 KD. Lower viscosity and higher solubility of low Mw Ch polymers makes them the appropriate candidates for preparing PECs. The polymers were completely dissolved in diluted acetic acid solution (pH ≈ 6) while Ch9 is even soluble in distilled water due to its low Mw.

CMD was thiolated by conjugating L-Cys to the polymer carboxyl groups via amide bonds. The comparison between FTIR spectra of TD and CMD demonstrates the efficient Cys conjugation (Fig. S1). The band intensity of carboxyl and carboxylate groups appearing at 1712 and 1608 reduced by increasing the rate of thiolation, while the band intensity of the amide bind enhanced at 1630. Ellman’s test also confirmed the sufficient thiolation of CMD, approximately 335±12 µmol/g of CMD.

**
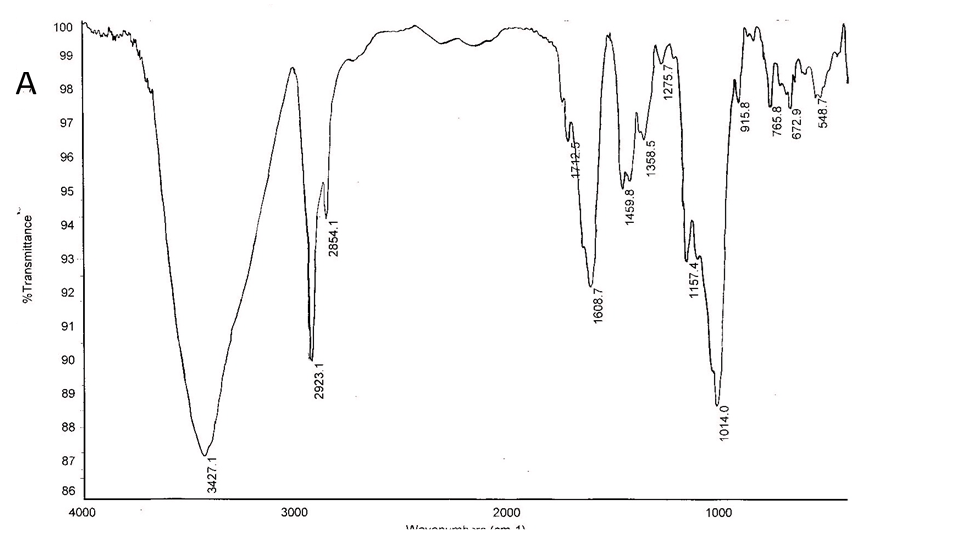
**

**
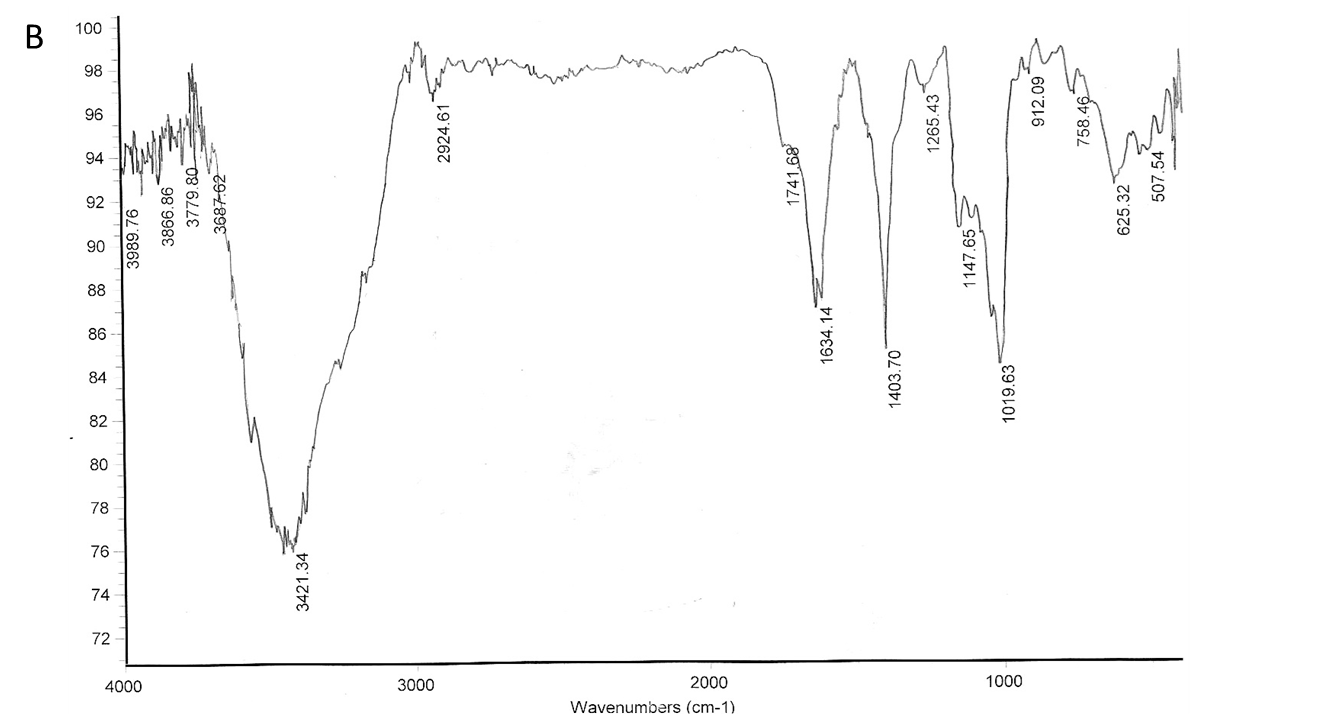
**

Fig. S1. FTIR spectra of carboxymethyl dextran (A) and thiolated dextran (B).

1. **TGA**

TGA was executed to confirm complexation of CMD and Ch. As illustrated in Fig. S1, two stages of mass loss were distinguished for Ch between 47.33 to 84.58 ^o^C with T_peak_ of 64.05 and between 227.48 and 296.98 ^o^C with T_peak_ of 259.21 ^o^C associated to the loss of water content (12.59%) and polymer decomposition (50.18%), respectively.

For CMD, three weight loss stages were detected as the first stage related to the loss of water content (10.33%) occurred between 51.57 to 134.37 ^o^C with T_peak_ of 91.82, and the other stages associated to the 63.562% polymer weight loss between 281.07 to 314.47 ^o^C with T_peak_ of 297.72 ^o^C, and 13.157% polymer weight loss between 540.95 to 549.29 ^o^C with T_peak_ of 546.49 ^o^C.

For the PEC of CMD and Ch with D/Ch ratio of 0.2, two stages of weight loss were perceived. The first one was between 32.91 to 63.70 ^o^C with 14.81% weight loss and T_peak_ of 48.75 ^o^C, that similar to the other samples, it related to the water content of the PECs. Weight loss of PECs due to the CMD or Ch decomposition (90.364%) occurred between 221.57 to 306.04 ^o^C with T_peak_ of 286.26 ^o^C which is between T_peak_ of Ch and CMD demonstrating complexation of the polymers by electrostatic interaction [43].


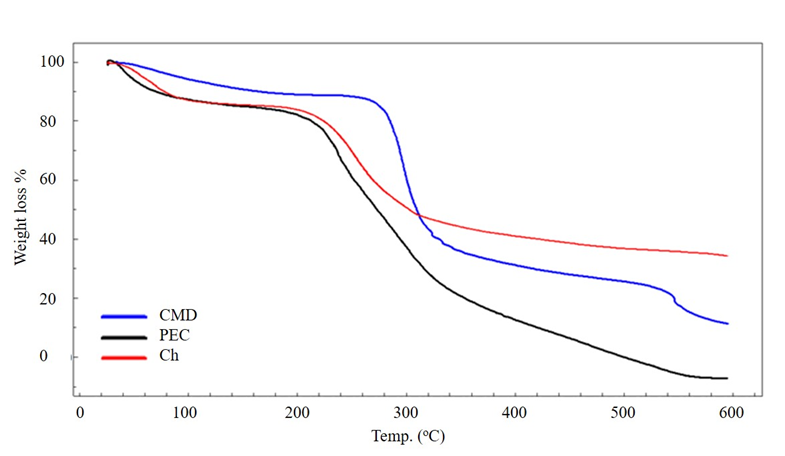


Fig. S2. Nano PEC characterization; (A) Thermogravimetric analysis of chitosan (Ch), carboxymethyl dextran (CMD), and polyelectrolyte complex (PEC) of chitosan 18 KD (Ch18) and CMD with CMD to Ch molar ratio of 0.2.

1. **Size distribution and zeta potential of nanoparticles**

The initial size of PECs could be influenced by parameters manipulating nanoparticle composition such as kind of dextran (CMD/TD), Ch Mw, and D/Ch ratio. Moreover, PECs are kind of nanohydrogels which absorb water when disperse in liquid medium and the hydrodynamic size increases following swelling of nanohydrogels [32]. Thus, not only PEC structure but also preparation medium composition can manipulate amount of PEC swelling and a consequent hydrodynamic size. As presented in table 3, nanoparticle size raised noticeably (p<0.05) by mounting D/Ch ratio as there is less electrostatic cross linking in PEC structure with lower amounts of Ch amine groups. Also, lower zeta potential of the PECs with D/Ch ratio of 5 enhances the rate of nanoparticle aggregation. The size of PECs was diminished by increasing Ch Mw, but it was not statistically significant for non-thiolated ones. Mw of CMD was about 10 KD almost same as Mw of Ch9. It seems that using Ch18, which has longer chains than dextran, the smaller compact PECs was obtained that enhanced the chance of collision of thiol groups and disulfide cross linking leading to the denser PECs. Moreover, the thiolated PECs were smaller than non-thiolated PECs due to the internal crosslinking that restricted the swelling of the PECs. Excluding PECs with D/Ch ratio of 5, the others had positive zeta potential since the surface of them was mostly covered by Ch amine groups. According to the CMD monomer Mw (220 g/mol), and amounts of carboxyl moieties per each gram of the polymer (1.1-1.5 mmol), the positive to negative charge ratios of the PECs with D/Ch ratios of 0.2, 1, and 5 are estimated 17.8, 3.6, and 0.7, respectively. Therefore, it was not unpredictable that zeta potential of the PECs with D/Ch ratio of 5 was negative, and by increasing Ch quantity, zeta potential was elevated.

1. **Acrylamide SDS-page electrophoresis**

The unedited SDS-page is shown in fig. S3.

**
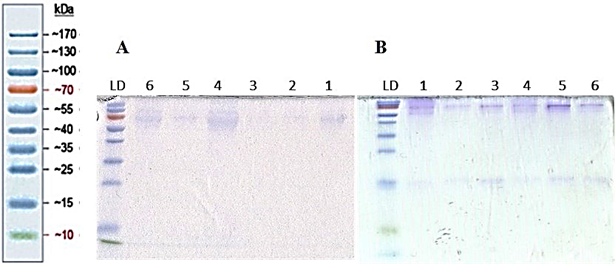
**

Fig. S3. SDS-page electrophoresis. Gel A shows the samples composed of TD and Gel B shows the ones composed of CMD. PECs of Ch9 with D/Ch ratio of 0.2 (1), with D/Ch ratio of 1 (2), with D/Ch ratio of 5 (3), PECs of Ch18 with D/Ch ratio of 0.2 (4), with D/Ch ratio of 1 (5), with D/Ch ratio of 5 (6).

1. **LC-MS/MS spectroscopy**

A


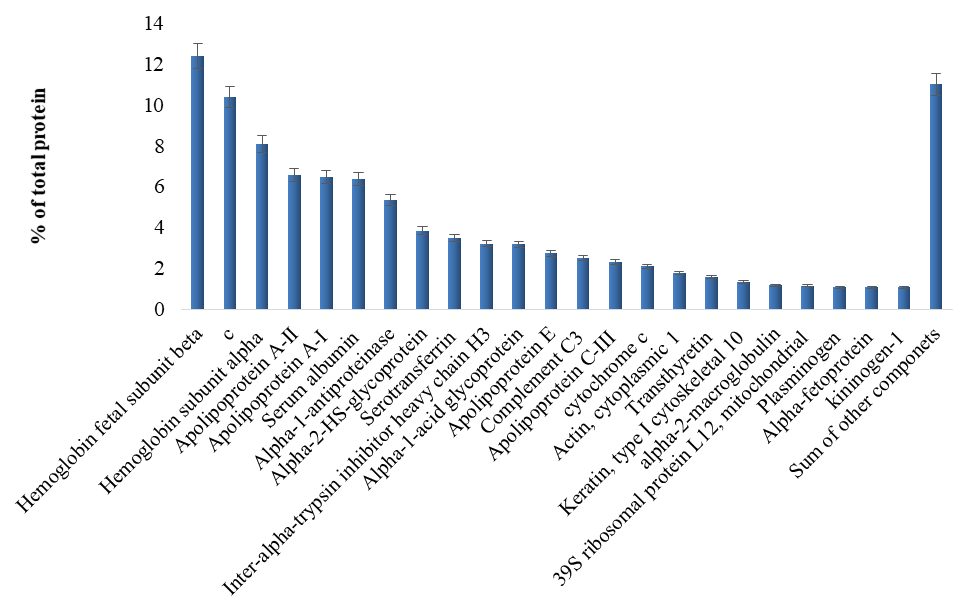


B


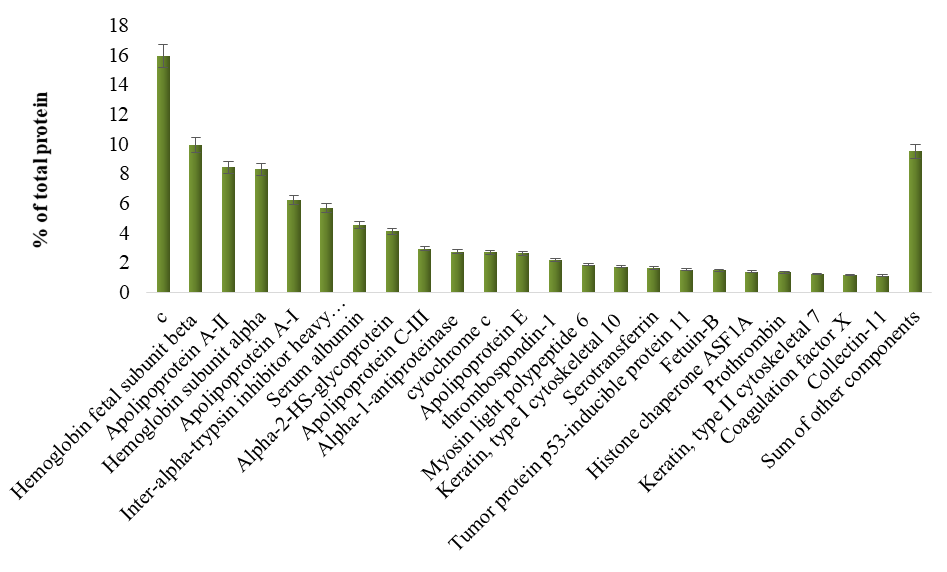


C


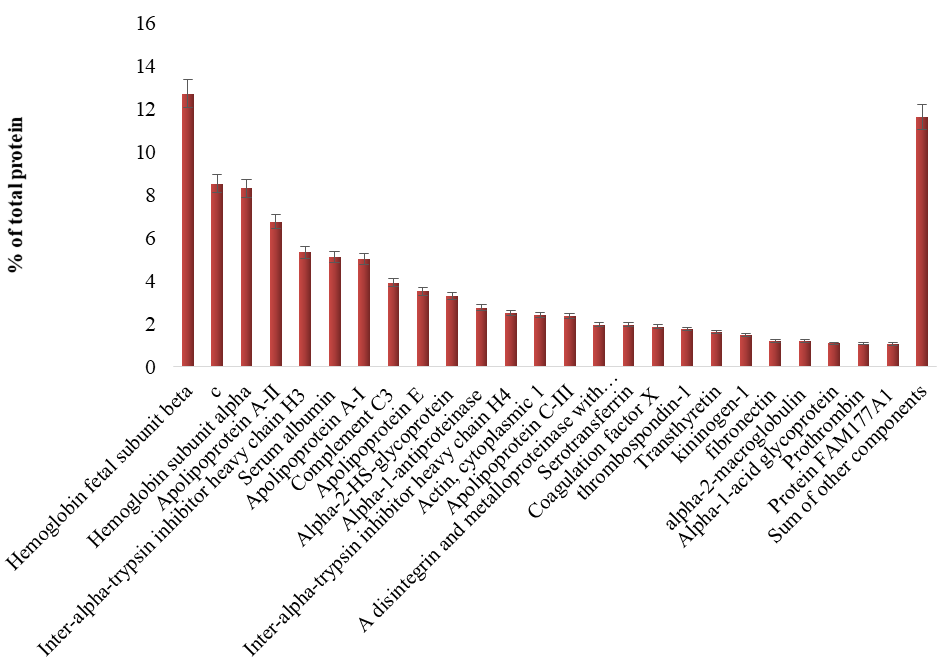


Fig.S4. Protein corona composition of different PECs incubated with FBS 30% obtained by LC-MS/MS analysis. The PECs of TD and Ch18 with D/Ch ratio of 0.2 (A), with D/Ch ratio of 5 (B), and the PECs of CMD and Ch18 with D/Ch ratio of 5 (C).

1. **Confocal microscopy**

**
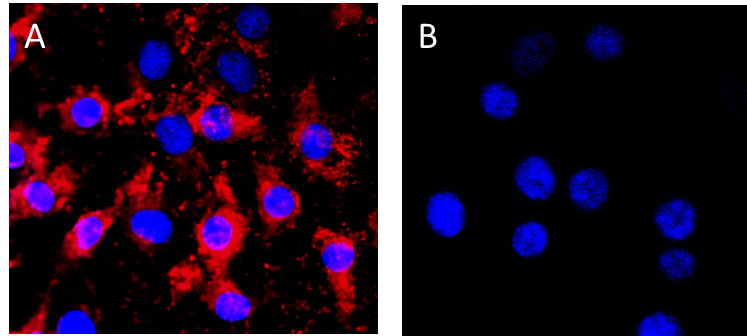
**

Fig. S5. Confocal microscopy of the cells treated with the PECs of CMD and Ch18 with D/Ch ratio of 5 (A) and negative control cells (B). The nuclei of the cells were stained by DAPI.

1. **Positron emission tomography/ computed tomography (PET/CT) study**

Maximum and mean activity concentrations (Bq/cc) for critical organs were measured in all PET images (Table S1).

Table S1. Maximum and mean activity concentrations (Bq/cc) for critical organs of rats

|  | |  | Heart | | activity concentrations (Bq/cc) in organs  Kidney | | Liver and Spleen | |
| --- | --- | --- | --- | --- | --- | --- | --- | --- |
| Scanning  time | animal group | | Mean | Max | Mean | Max | Mean | Max |
| 30 min | Group 1 | | 0.749± 0.121 | 1.102± 0.218 | 2.390± 0.167 | 11.02± 0.871 | 0.596± 0.062 | 0.751± 0.038 |
|  | Group 2 | | 0.464± 0.052 | 0.782± 0.052 | 1.977± 0.334 | 6.012± 0.722 | 0.356± 0.015 | 0.511± 0.029 |
|  | Group 3 | | 0.165± 0.004 | 0.217± 0.011 | 0.561± 0.024 | 1.897± 0.023 | 0.120± 0.004 | 0.155± 0.018 |
| 60 min | Group 1 | | 0.831± 0.094 | 1.236± 0.212 | 5.633± 0.319 | 23.68± 1.869 | 0.718± 0.057 | 0.914± 0.047 |
|  | Group 2 | | 0.601± 0.113 | 1.008± 0.213 | 1.350± 0.171 | 3.795± 0.255 | 0.472± 0.051 | 0.695± 0.020 |
|  | Group 3 | | 0.669± 0.050 | 0.492± 0.051 | 1.147± 0.024 | 5.870± 0.311 | 0.225± 0.006 | 0.293± 0.009 |
| 120 min | Group 1 | | 0.202± 0.032 | 0.271± 0.067 | 1.434± 0.083 | 6.041± 0.501 | 0.241± 0.012 | 0.507± 0.044 |
|  | Group 2 | | 0.176± 0.017 | 0.299± 0.008 | 0.382± 0.011 | 1.301±  0.046 | 0.159± 0.012 | 0.221± 0.013 |
|  | Group 3 | | 0.183± 0.024 | 0.238± 0.014 | 0.597± 0.038 | 2.037± 0.173 | 0.123± 0.022 | 0.157± 0.006 |

^1^ *In vivo* bidistribution of the nanoparticles including the PECs composed of Ch18 and CMD with D/Ch ratios of 5 (group 1), Ch18 and TD with D/Ch ratios of 0.2 (group 2) and 5 (group 3).
